# MDA-7/IL-24 functions as a tumor suppressor gene *in vivo* in transgenic mouse models of breast cancer

**DOI:** 10.18632/oncotarget.6047

**Published:** 2015-10-12

**Authors:** Mitchell E. Menezes, Xue-Ning Shen, Swadesh K. Das, Luni Emdad, Chunqing Guo, Fang Yuan, You-Jun Li, Michael C. Archer, Eldad Zacksenhaus, Jolene J. Windle, Mark A. Subler, Yaacov Ben-David, Devanand Sarkar, Xiang-Yang Wang, Paul B. Fisher

**Affiliations:** ^1^ Department of Human and Molecular Genetics, Virginia Commonwealth University, School of Medicine, Richmond, Virginia, USA; ^2^ VCU Institute of Molecular Medicine, Virginia Commonwealth University, School of Medicine, Richmond, Virginia, USA; ^3^ VCU Massey Cancer Center, Virginia Commonwealth University, School of Medicine, Richmond, Virginia, USA; ^4^ Department of Anatomy, Norman Bethune College of Medicine, Jilin University, Changchun, China; ^5^ Departments of Medical Biophysics, University of Toronto, Ontario, Canada; ^6^ Nutritional Sciences, University of Toronto, Ontario, Canada; ^7^ Toronto General Research Institute - University Health Network, Toronto, Ontario, Canada; ^8^ Division of Biology, the Key Laboratory of Chemistry for Natural Products of Guizhou Province and Chinese Academy of Sciences, Guiyang, China

**Keywords:** melanoma differentiation associated gene-7/interleukin-24 (MDA-7/IL-24), MMTV-PyMT mice, MMTV-*MDA-7* mice, MMTV-*MDA-7*/MMTV-*Erbb2* mice, transgenic mice

## Abstract

Melanoma differentiation associated gene-7/Interleukin-24 (MDA-7/IL-24) is a novel member of the IL-10 gene family that selectively induces apoptosis and toxic autophagy in a broad spectrum of human cancers, including breast cancer, without harming normal cells or tissues. The ability to investigate the critical events underlying cancer initiation and progression, as well as the capacity to test the efficacy of novel therapeutics, has been significantly advanced by the development of genetically engineered mice (GEMs) that accurately recapitulate specific human cancers. We utilized three transgenic mouse models to better comprehend the *in vivo* role of MDA-7/IL-24 in breast cancer. Using the MMTV-PyMT spontaneous mammary tumor model, we confirmed that exogenously introducing MDA-7/IL-24 using a *Cancer Terminator Virus* caused a reduction in tumor burden and also produced an antitumor “bystander” effect. Next we performed xenograft studies in a newly created MMTV-*MDA-7* transgenic model that over-expresses MDA-7/IL-24 in the mammary glands during pregnancy and lactation, and found that MDA-7/IL-24 overexpression delayed tumor growth following orthotopic injection of a murine PDX tumor cell line (mPDX) derived from a tumor formed in an MMTV-PyMT mouse. We also crossed the MMTV-*MDA-7* line to MMTV-*Erbb2* transgenic mice and found that MDA-7/IL-24 overexpression delayed the onset of mammary tumor development in this model of spontaneous mammary tumorigenesis as well. Finally, we assessed the role of MDA-7/IL-24 in immune regulation, which can potentially contribute to tumor suppression *in vivo*. Our findings provide further direct *in vivo* evidence for the role of MDA-7/IL-24 in tumor suppression in breast cancer in immune-competent transgenic mice.

## INTRODUCTION

Breast cancer remains one of the most commonly diagnosed cancers among women and is the second leading cause of cancer-related death in the United States [American Cancer Society, Cancer Facts & Figures, 2015]. Increased awareness about breast cancer has aided early diagnosis of the disease in recent years; however patient prognosis is adversely affected once breast cancer metastasizes to distant areas in the body. Hence, there is an urgent need to delineate the mechanisms regulating breast cancer development, progression and metastasis in order to develop novel targeted therapeutics with enhanced potency. While *in vitro* tissue culture studies and *in vivo* xenograft studies in immunocompromised mice have expanded our understanding of breast cancer, transgenic mouse models harboring intact functional immune systems provide additional advantages and are indispensible tools for studying breast cancer [1-2]. By utilizing mammary-specific promoters that drive tumor-promoting genes, researchers have developed transgenic mouse lines that spontaneously develop mammary tumors [3-4]. Knockout and conditional knockout lines have similarly been developed to better understand the role of tumor suppressor genes in the mammary gland [2]. In these contexts, genetically modified mice have helped identify genes involved in tumor initiation, progression and metastasis and provide valuable tools to assess novel therapeutics for human breast cancer.

Melanoma differentiation associated gene-7 (*mda-7*) [5-6], also known as Interleukin-24 *(IL-24*) [7-8], encodes a secreted protein of the IL-10 gene family and is located on chromosome 1q32-33 in humans [9-10]. At normal physiological levels, MDA-7/IL-24 functions as a cytokine and is expressed in tissues of the immune system such as the thymus, spleen, peripheral blood leukocytes (PBL) and normal melanocytes [9, 11]. MDA-7/IL-24 was also found to play a role in wound healing [12], in autoimmune diseases [13] and provided protection against a number of infectious bacteria including *Pseudomonas aeruginosa* [14], *Salmonella typhimurium* [15] and *Mycobacterium tuberculosis* [16]. At supra-physiological levels, MDA-7/IL-24 displays anti-cancer properties towards breast cancer including inhibition of tumor growth, invasion, metastasis, angiogenesis and tumor-initiating/stem cells [17-21]. Our previous studies in breast cancer, utilizing a conditionally replication-competent adenovirus expressing *mda-7/IL-24* (also known as a cancer terminator virus - *CTV*) showed that MDA-7/IL-24 could efficiently target primary as well as distant breast carcinomas for elimination in athymic mice [20-21]. Importantly, MDA-7/IL-24 was shown to be non-toxic to normal cells [6, 20-27]. Our recent study showed that MDA-7/IL-24 also inhibited the growth and self-renewal potential of breast cancer-initiating/stem cells without any adverse effects on normal breast stem cells [17]. The role and mechanism of action of MDA-7/IL-24 in tissue culture and athymic xenograft models of breast cancer have previously been studied [19-27]; however few studies evaluated the role of MDA-7/IL-24 in an immune competent transgenic model [28-30].

To evaluate the role of MDA-7/IL-24 in breast cancer in immune-competent mice and to gain a further understanding of the mechanism of action of MDA-7/IL-24 in breast cancer, we performed *in vivo* experiments using three transgenic models - MMTV-PyMT mice, MMTV-*MDA-7* mice and MMTV-*MDA-7*/MMTV-*Erbb2* mice. Our results illustrate that MDA-7/IL-24 delayed tumor onset, suppressed tumor growth and also had anti-tumor “bystander” effects. Further, we also found that MDA-7/IL-24 mounted an antitumor immune response by increasing levels of infiltrating CD8^+^ T cells and the frequency of IFN-γ or granzyme B-producing CD8^+^ T cells in the mammary tumors. Accordingly, our findings confirm that MDA-7/IL-24 is a relevant therapeutic option even in immune competent mice, both in xenograft models and spontaneous tumor models, and can synergize with the immune system to directly target tumor cells for destruction.

## RESULTS

### MDA-7/IL-24 reduces tumor growth in MMTV-PyMT transgenic mice

**Figure 1:**
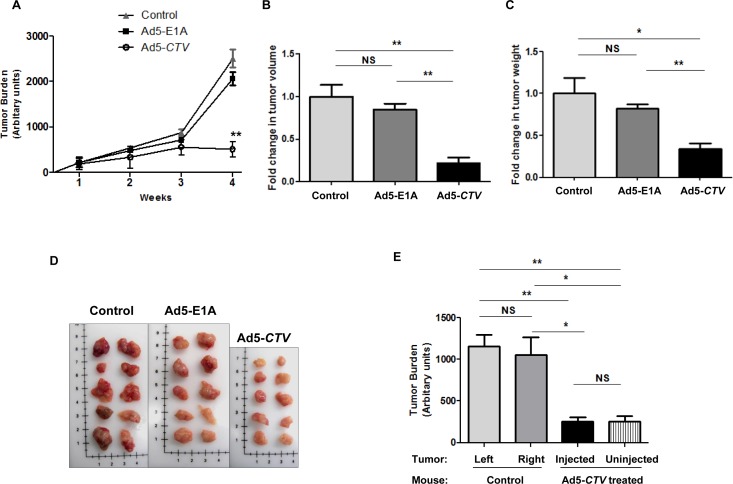
MDA-7/IL-24 inhibits tumor growth in MMTV-PyMT transgenic mice **A.** Mice treated intratumorally with Ad5-E1A, Ad5-*CTV* or untreated controls were monitored for tumor burden over 4 weeks following first appearance of tumors. Tumor burden is represented in arbitrary units. **B.** Fold-change in total tumor volume in mice receiving Ad5-E1A, Ad5-*CTV* and control untreated mice. **C.** Fold-change in total tumor weight in mice receiving Ad5-E1A, Ad5-*CTV* and control untreated mice. **D.** Representative images of total tumors present in MMTV-PyMT mice treated with Ad5-E1A, Ad5-*CTV* and untreated controls at the time of sacrifice. Image dimensions are approximately adjusted to match the scale in each image. E. Comparison of tumor burden in control untreated mice (tumors from left and right sides) and Ad5-*CTV*-treated mice (injected and uninjected tumors). Uninjected tumors in mice receiving Ad5-*CTV* also showed a decrease in tumor size, indicating an anti-tumor “bystander” activity. *, *p* < 0.05; **, *p* < 0.01; NS, not significant.

**Figure 2:**
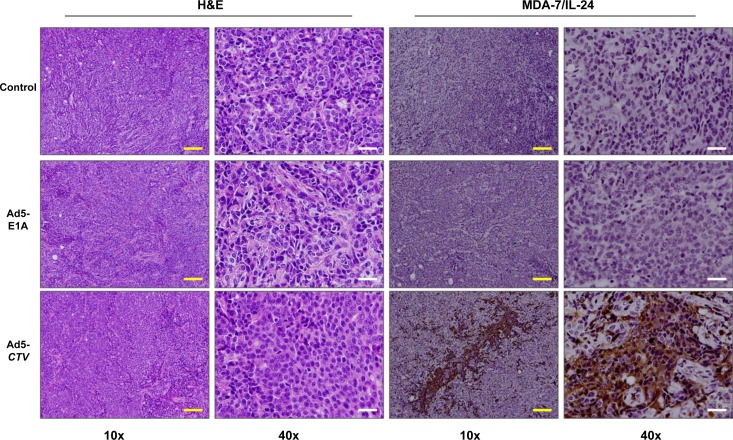
Ad5-*CTV* leads to robust expression of MDA-7/IL-24 in Ad5-*CTV* injected tumors in MMTV-PyMT mice Ad5-E1A, Ad5-*CTV* and control untreated tumors were sectioned and immunohistochemistry was performed for H&E staining and to assess MDA-7/IL-24 expression. As would be expected, tumors injected with Ad5-*CTV* showed MDA-7/IL-24 expression. Yellow bars = 100 μm, white bars = 20 μm.

To evaluate the relevance of MDA-7/IL-24 in suppression of tumor growth in immune competent mice, we initially utilized the MMTV-PyMT transgenic mouse model [4]. Female MMTV-PyMT transgenic mice develop tumors in all mammary glands within 2 to 3 months of age [4]. In this model, we used a tumor-specific conditionally replicating virus expressing MDA-7/IL-24 (designated a cancer terminator virus or *CTV*) [31]. A type 5 adenovirus (Ad5) was engineered to specifically replicate in tumor cells by expressing Ad5-E1A under the control of a cancer-specific promoter derived from progression elevated gene-3 [32-33] and also produces MDA-7/IL-24 (Ad5-*CTV*) [20, 23-24, 29-30]. As a control, we included Ad5-E1A that also replicates in tumor cells, but lacks MDA-7/IL-24. Untreated mice served as an additional control to evaluate tumor development and progression in MMTV-PyMT mice. The mice were monitored for tumor onset and tumors were injected with the respective virus as described in the Materials and Methods once palpable tumors were observed. Mice that were injected intratumorally with Ad5-*CTV* showed a significant reduction in tumor burden as compared to mice treated intratumorally with Ad5-E1A or control untreated mice (Figure 1A). Additionally, the total tumor burden at harvest was significantly reduced in mice treated with Ad5-*CTV* as compared to the tumor burden in mice treated with Ad5-*E1A* or untreated mice (Figure 1B to 1D). In order to evaluate both the tumor suppressive effects of MDA-7/IL-24 as well as the “bystander” anti-tumor effect of MDA-7/IL-24, approximately 50% of the tumors that formed were injected with the respective virus, while the remaining 50% were left uninjected. Interestingly, the tumors that were left untreated on mice with 50% tumors treated with Ad5-*CTV* also showed a reduction in tumor growth, indicating a “bystander” anti-tumor effect (Figure 1E). Immunohistochemical staining showed that MDA-7/IL-24 was expressed in the Ad5-*CTV* injected tumors (Figure 2) as well as the untreated tumors in the same mouse (Supplemental Figure 1A). As would be expected, MDA-7/IL-24 expression was higher in the injected tumors as compared to the uninjected tumors in the same mouse (Supplemental Figure 1A).

### Tumor growth is reduced in MMTV-*MDA-7* transgenic mice

**Figure 3:**
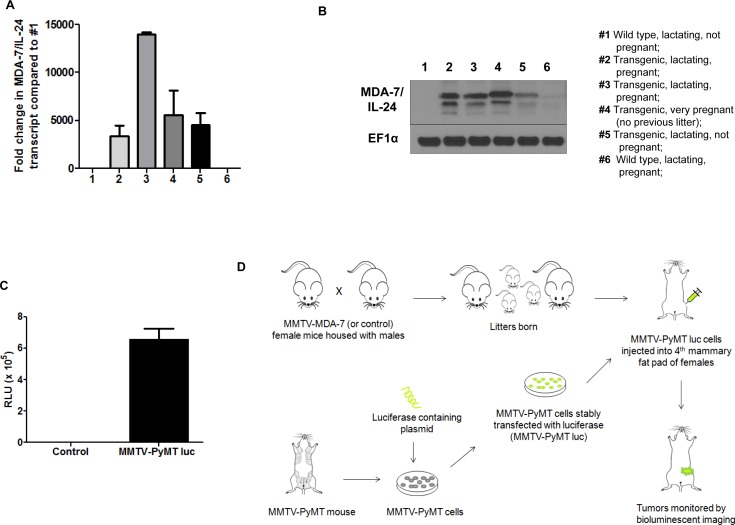
MDA-7/IL-24 is robustly expressed in MMTV-*MDA-7* transgenic mice during pregnancy and lactation Mammary fat pads were harvested from MMTV-*MDA-7* (positive and negative/control/non-transgenic littermate) pregnant, and lactating mice and protein and RNA were extracted. MDA-7/IL-24 expression was assessed in the mammary fat pad of MMTV-*MDA-7* transgenic mice at the transcript **A.** and protein **B.** levels. **C.** Tumor cells were harvested from MMTV-PyMT mice bearing tumors and grown *in vitro*. These cells were stably transfected with a luciferase-expressing construct to generate MMTV-PyMT luc cells. Luciferase expression was assessed in these MMTV-PyMT luc (mPDX luc) cells. **D.** Schematic of the xenograft study in MMTV-*MDA-7* transgenic mice. Female (MMTV-*MDA-7* positive and negative/control/non-transgenic littermates) and male mice were housed together to allow for offspring. Following the birth of first litter, MMTV-PyMT luc cells were injected into the 4^th^ mammary fat pad of the female mice and tumor growth was monitored by bioluminescent imaging.

**Figure 4:**
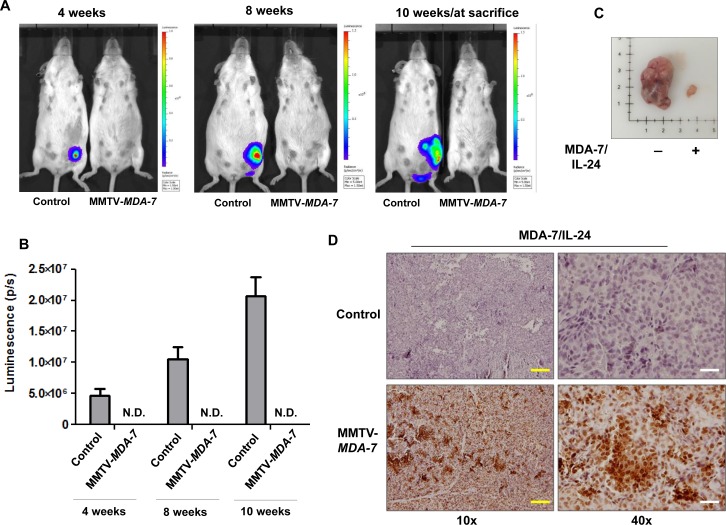
MDA-7/IL-24 suppresses tumor growth in MMTV-*MDA-7* transgenic mice **A.** Representative bioluminescent images at 4, 8 and 10-weeks post injection of MMTV-PyMT luc (mPDX luc) cells into the 4^th^ mammary fat pad of female MMTV-*MDA-7* negative (control) and positive mice. **B.** Graphical representation of the quantification of luminescence signals. IVIS spectrum coupled with Living Image 4.3.1 was used for the quantification. MMTV-*MDA-7* mice showed no detectable luminescence signal when imaged alongside the control non-transgenic mice, (N.D. = not detected). **C.** Images of tumors from MMTV-*MDA-7* negative (control/non-transgenic littermate) and positive mice at sacrifice. D. Immunohistochemistry showing expression of MDA-7/IL-24 in tumor sections of MMTV-*MDA-7* positive but not MMTV-*MDA-7* negative (control/non-transgenic littermate) mice. Yellow bars = 100 μm, white bars = 20 μm.

Next we developed MMTV-*MDA-7* transgenic mice that specifically express *mda-7/IL-24* under the transcriptional control of the MMTV LTR (mouse mammary tumor virus long terminal repeat) promoter. Glucocorticoid, androgen and progesterone response elements are present within the MMTV LTR promoter and are regulated by the estrous cycle in the mammary glands. Transcription of genes under the control of the MMTV LTR promoter are highly up regulated during pregnancy and peak during lactation [34]. To validate expression of MDA-7/IL-24 within the mammary glands of MMTV-*MDA-7* mice, we harvested mammary fat pads from pregnant and lactating mice, extracted protein and RNA, and evaluated the expression of MDA-7/IL-24. As would be expected with the MMTV promoter, MDA-7/IL-24 was robustly expressed during pregnancy and lactation both at the transcript and protein level (Figure 3A and 3B). We did not observe an exact correlation between MDA-7/IL-24 expression at the transcript and protein level and this might be due to post translational modifications of the MDA-7/IL-24 protein. Regardless, we observed that MDA-7/IL-24 was up regulated during pregnancy and lactation. Next, we established MMTV-PyMT cells from MMTV-PyMT mammary tumors, a murine PDX (patient derived xenograft) (mPDX) cell line, and stably transfected the cells with a luciferase-expressing plasmid in order to monitor tumor growth using bioluminescence (MMTV-PyMT luc cells) (Figure 3C). Since MDA-7/IL-24 is robustly expressed during lactation, MMTV-PyMT luc cells were introduced into the fourth mammary fat pad approximately 20 days after the birth of the first litter (Figure 3D). Mice were monitored for tumor growth using bioluminescence imaging [33, 35] (Figure 4A and 4B). In order to maintain MDA-7/IL-24 levels within the mammary glands, females were housed continuously with males to allow for normal pregnancy and lactation cycles. Non-transgenic littermate females were used as controls (MMTV-*MDA-7* negative mice). The control mice showed tumor formation by 4 weeks following introduction of tumor cells into the mammary fat pad, however, MMTV-*MDA-7* mice showed delayed tumor onset. The difference in tumors in MMTV-*MDA-7* and control mice at 10 weeks was dramatic (Figure 4). Immunohistochemical findings show that MDA-7/IL-24 was expressed in the MMTV-*MDA-7* transgenic mouse tumors (Figure 4D).

### MMTV-*MDA-7*/MMTV-*Erbb2* compound transgenic mice show delayed tumor onset

**Figure 5:**
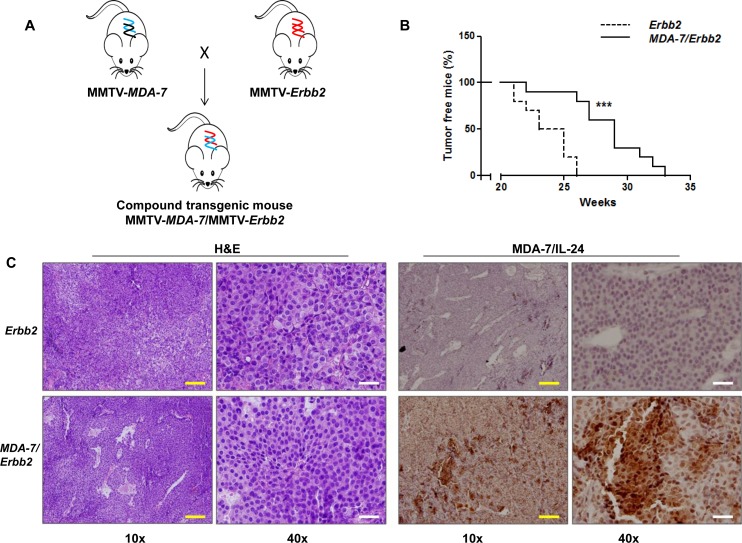
Tumor onset is delayed in MMTV-*MDA-7*/MMTV-*Erbb2* compound transgenic mice **A.** Schematic of generation of MMTV-*MDA-7*/MMTV-*Erbb2* compound transgenic mice. MMTV-*MDA-7* hemizygous mice were mated with MMTV-*Erbb2* homozygous mice. **B.** Kaplan Meier analysis showing delay in tumor onset in MMTV-*MDA-7*/MMTV-*Erbb2* compound transgenic mice. **C.** Immunohistochemistry showing H&E staining and MDA-7/IL-24 expression in tumor sections of MMTV-*Erbb2* and MMTV-*MDA-7*/MMTV-*Erbb2* mice. Yellow bars = 100 μm, white bars = 20 μm. ***, *p* < 0.001.

Finally, to determine the relevance of the presence of MDA-7/IL-24 on tumor onset and progression of a model of spontaneous mammary tumor development, we generated MMTV-*MDA-7*/MMTV-*Erbb2* compound transgenic mice (Figure 5A). MMTV-*Erbb2* transgenic mice develop mammary tumors spontaneously over a 5-8 month period [3, 36]. We decided to use the MMTV-*Erbb2* model for our study because we wanted to determine the role of MDA-7/IL-24 in a model that develops tumors over a prolonged period of time. MMTV-PyMT transgenic mice are invaluable in understanding tumor onset and progression. However, because of the robust and aggressive nature of the tumors that form and the relatively short life span of these mice (about 4 months), we decided to use the MMTV-*Erbb2* mouse model with comparatively slower tumor kinetics. In this model, both *Erbb2* and *mda-7/IL-24* transgene expression is dependent on pregnancy and lactation, so the females were again housed continuously with males to allow for normal pregnancy and lactation cycles. In this model, we observed a significant delay in tumor onset in the compound transgenic mice expressing both *mda-7/IL-24* and *Erbb2* transgenes as compared to the MMTV-*Erbb2* littermate controls (Figure 5B). Immunohistochemical staining showed that MDA-7/IL-24 was expressed in the MMTV-*MDA-7*/MMTV-*Erbb2* compound mouse tumors (Figure 5C).

### MDA-7/IL-24 regulates antitumor immune response to facilitate tumor suppression in MMTV-PyMT transgenic mice

**Figure 6:**
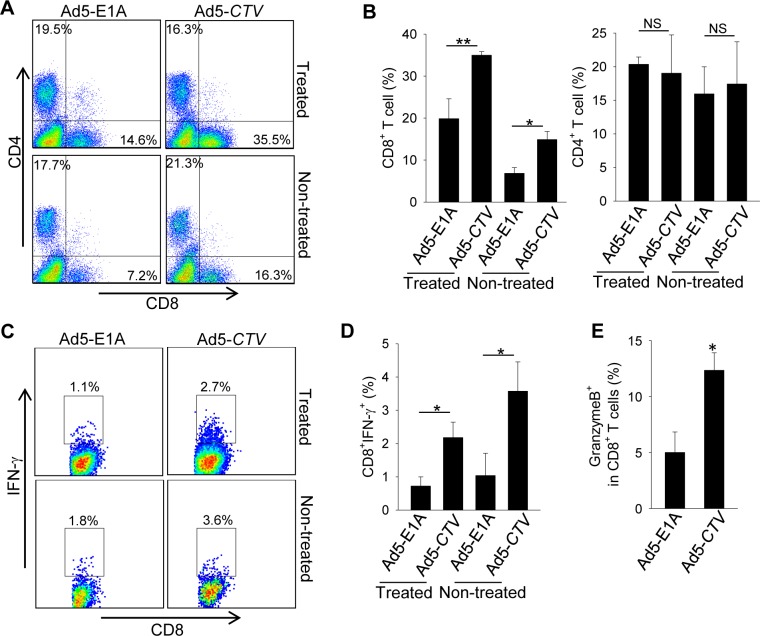
MDA-7/IL-24 enhances anti-tumor immune responses against MMTV-PyMT mammary tumors Mammary tumors of MMTV-PyMT transgenic mice were treated with Ad5-*CTV* or Ad5-E1A as a control. **A.** and **B.** CD8^+^ and CD4^+^ T cell infiltration in the treated tumors and non-treated tumors were analyzed by FACS. The frequency in panel A is calculated as the ratio of CD8^+^ or CD4^+^ T cells among all the cells in the tumors. **C.** to **E.** Frequency of IFN-γ or granzyme B producing CD8^+^ T cells was assayed by intracellular IFN-γ staining and FACS. The numbers in panel C are the frequency of IFN-γ producing cells among CD8^+^ or CD4^+^ T cells. *, *p* < 0.05; **, *p* < 0.01; NS, not significant.

Recent studies have highlighted the importance of the immune system in regulation of cancer development and progression, including breast cancer [37-39]. Since most other experimental models that assessed the relevance of MDA-7/IL-24 in breast cancer did not have an intact immune system, we examined immune infiltrates in the tumors to determine the potential relevance of an intact immune system in MDA-7/IL-24-mediated tumor suppression. Our results show that intratumoral injection of Ad5-*CTV* resulted in a marked increase in tumor infiltrating CD8^+^ T cells compared to Ad5-E1A treatment (Figure 6A and 6B). These CD8^+^ T cells from Ad5-*CTV*-treated tumors displayed higher levels of IFN-γ expression than those from Ad5-E1A treated tumors, as determined by intracellular cytokine staining (Figure 6C and 6D). Although the recruitment of CD4^+^ T cells was similar in these two groups, the infiltrating CD4^+^ T cells from Ad5-*CTV* treated tumors also produced more IFN-γ (data not shown). In addition, a significant increase in infiltrating CD8^+^ T cells, as well as expression of IFN-γ and granzyme B by CD8^+^ T cells was observed in non-treated tumors derived from MMTV-PyMT transgenic mice that received Ad5-*CTV* (Figure 6A to 6E), suggesting that Ad5-*CTV* treatment induced a systemic immune response against all the mammary tumors within the MMTV-PyMT transgenic mouse model.

## DISCUSSION

Several research groups including our own have shown that MDA-7/IL-24 plays a tumor inhibitory role in several cancers including breast cancer [17, 20-21], prostate cancer [6, 24, 28-30, 40], melanoma [5-6, 23, 27], ovarian cancer [41-42], colorectal cancer [43-46], pancreatic cancer [47-49], non-small cell lung carcinoma [50-52], glioblastoma [53-55], hepatocellular cancers [56-57], and nasopharyngeal cancers [58]. MDA-7/IL-24 functions at multiple levels to promote anti-cancer properties. MDA-7/IL-24 induces cell death *via* apoptosis and/or toxic autophagy, inhibition of invasion and metastasis, “bystander” anti-cancer activity, radiosensitization, anti-angiogenesis and cancer initiating/stem cell killing [7-8, 59].

*In vitro* studies showed that MDA-7/IL-24 induced G_2_/M cell cycle arrest in breast cancer cells *via* downregulation of AKT-GSK3β and upregulation of cyclin-dependent kinase inhibitor and apoptosis by activation of caspase-dependent signaling pathways and BAX signaling [21, 60-61]. However, normal breast cells were not adversely affected. MDA-7/IL-24 expression also inhibited tumor development in athymic xenograft mouse models [20-21]. Further studies showed that MDA-7/IL-24 could interact with BiP/GRP78 and activate p38 MAPK and GADD expression to selectively cause apoptosis in cancer cells [62-63]. Importantly, MDA-7/IL-24 also inhibited tumor growth of distant untreated tumors in the same athymic mouse, indicating MDA-7/IL-24 also had “bystander” anti-tumor activity [20]. Studies using rat mammary tumor models also showed that MDA-7/IL-24 could efficiently suppress tumor development [18]. MDA-7/IL-24 induced the expression of growth arrest-specific gene-3 (GAS3). GAS3 interacts with β1 integrin and disrupts the interaction between β1 integrin and fibronectin, leading to suppression of mammary tumor growth [18]. Recent studies in breast cancer-initiating/stem cells showed that MDA-7/IL-24 induced apoptosis and endoplasmic reticulum stress and inhibited self-renewal potential of breast cancer-initiating/stem cells by suppressing the Wnt signaling pathway [17]. Thus, while the mode of action and role of MDA-7/IL-24 in mammary tumors has been well studied, the relevance of MDA-7/IL-24 in an immune competent animal is not well defined.

In our study, we evaluated the relevance of MDA-7/IL-24 in transgenic models with an intact immune system. The immune system plays a very important role in regulating tumor growth and progression and hence understanding the role of MDA-7/IL-24 in immune competent mice is important. Our first strategy was to introduce MDA-7/IL-24 by adenoviral injection into mammary tumors arising spontaneously in MMTV-PyMT transgenic mice. As expected, introduction of MDA-7/IL-24 caused a reduction in the growth of the injected tumors. Importantly, MDA-7/IL-24 also showed anti-cancer “bystander” activity, since the growth of uninjected tumors was also suppressed in animals in which tumors were injected with the MDA-7/IL-24-expressing virus. When tumor sections were assessed for MDA-7/IL-24 expression by immunohistochemistry, we observed that both injected and uninjected tumors in the mice showed the presence of MDA-7/IL-24. Presence of MDA-7/IL-24 in the injected tumors validated that Ad5-*CTV* could replicate within the mammary tumors. Presence of MDA-7/IL-24 in the uninjected tumors, albeit at lower levels, suggests that MDA-7/IL-24 secreted by the injected tumors might have entered the circulation and localized at the distant tumor site, or more likely the secreted protein interacted with IL-20/IL-22 receptors on the distant tumor, inducing MDA-7/IL-24 production through a “paracrine/autocrine” effect [22] thereby mediating tumor reduction. Alternatively, production of progeny virus might have entered the circulation and caused secondary infection of distant tumors, resulting in production of MDA-7/IL-24. This would appear less likely, since after several injections of the Ad5-*CTV* directly into tumors it is predicted that the newly released virus would enter the circulation and either be trapped in the liver or eliminated by the immune system (Supplemental Figure 1B).

Our second strategy was to xenograft cells isolated from mammary tumors developing in MMTV-PyMT (mPDX) mice, akin to PDX tumors isolated from a patient, into the mammary fat pads of naïve MMTV-*MDA-7* mice. We introduced MMTV-PyMT luc (mPDX luc) cells into the mammary fat pads of mice after the MMTV-*MDA-7* mice had given birth to litters to ensure adequate MDA-7/IL-24 expression. Additionally, by housing males continuously with females and allowing for further offspring, we were able to maintain MDA-7/IL-24 expression within the mammary glands. This model showed a dramatic delay in tumor growth as compared to the other two models. This might be because we were able to control tumor development by xenografting mouse PDX tumor cells after MDA-7/IL-24 expression was present in the mammary glands. The mechanism underlying this suppression of PDX tumor growth in these mice and whether induction of apoptosis in a portion of the injected murine mPDX tumor cells contributed to the reduced final tumor volume requires further investigation.

Our third strategy was to co-express both a tumor promoting gene, *Erbb2*, and MDA-7/IL-24 endogenously in the mammary gland, and to follow tumor progression. Since the MMTV-PyMT mouse model is a very aggressive model and tumors form even in virgin mice [4], while MDA-7/IL-24 is optimally expressed during pregnancy and lactation in MMTV-*MDA-7* mice, we decided to use the MMTV-*Erbb2* transgenic mouse model [4, 64] instead of the MMTV-PyMT model to generate the double transgenic animals. The *Erbb2* transgene expression is also regulated by pregnancy and lactation, and hence both Erbb2 and MDA-7/IL-24 expression would occur simultaneously. In this compound transgenic model, tumor onset was delayed by the presence of MDA-7/IL-24. Unlike previous results in athymic mice, we did not observe a complete regression of tumors in our models [20-21]. Given the current understanding of the role of MDA-7/IL-24, it was not expected that MDA-7/IL-24 alone would bring about a complete regression of tumors in transgenic mice as a single agent, since this effect would be dose-dependent even with viruses delivering higher amounts of MDA-7/IL-24. Additionally, in the absence of an intact immune system in athymic nude mice we anticipated enhanced efficacy of the administered Ad5-*CTV*, since it would be predicted that this conditionally replication competent virus would not be as efficiently cleared by the non-intact immune system in these animals. Of note, in preliminary unpublished studies combining Ad5-*CTV* with an Mcl-1 inhibitor (designated BI97-D6) resulted in enhanced anticancer activity in the MMTV-PyMT model (unpublished data and Supplemental Figure 2). Thus, our results represent important proof-of-principle studies regarding the relevance of MDA-7/IL-24 in immune competent animals and suggest that MDA-7/IL-24 might serve as an appropriate anti-cancer agent in combination with other therapeutics. Further, we assessed the role of MDA-7/IL-24 in engaging the immune system to mount an anti-tumor immune response against mammary tumors. Intratumoral injection of MDA-7/IL-24 caused an increase in tumor infiltrating, IFN-γ-producing CD8^+^ T cells. This enhanced immune activation was present in MDA-7/IL-24 treated tumors as well as non-treated tumors, indicating that a systemic antitumor immunity augmented by MDA-7/IL-24 may also contribute to its therapeutic activity in this breast cancer transgenic mouse model. Our findings, together with previous observations of MDA-7/IL-24 in promoting T-helper 1 (Th1) cytokines [11, 65-66], suggest that an immunostimulatory effect of MDA-7/IL-24 should be exploited for effective eradication of breast cancer.

The findings in an accompanying paper by Li et al. provide further evidence for the importance of MDA-7/IL-24 in mammary tumorigenesis (67). Using a mammary gland specific, tet-inducible MDA-7/IL-24 transgenic mouse model crossed with MMTV-Her2/Neu transgenic mice, the authors show that MDA-7/IL-24 caused an inhibition of tumor development. Additionally using a pre-existing tumor model by implanting lineage-depleted tumor cells isolated from MMTV-rtTA:IL24^tet-on^:MMTV-Her2/neu they show that MDA-7/IL-24 expression following doxycycline treatment significantly inhibited pre-existing tumor growth. They provided mechanistic insight into the signaling mechanism of MDA-7/IL-24 and show that the tumor suppressive effects observed in HER2^+^ breast cancer cells were mediated through PERP, a member of the GAS-3/PMP-22 family of tumor suppressors. The findings in the paper by Li et al. thus further establish the role of MDA-7/IL-24 in suppression of mammary tumors.

In conclusion, our study shows that MDA-7/IL-24 can delay tumor onset as well as tumor progression in transgenic mice and contributes to an immune response against mammary tumors. These important studies provide further *in vivo* evidence of the tumor suppressor function of this novel member of the IL-10 cytokine gene family [8, 10, 59]. Further studies are required to evaluate the combinatorial effect of MDA-7/IL-24 with immune components and other therapeutic agents, including chemotherapy, radiotherapy and antibody-based therapies in the context of animal models with intact immune systems. Studies using pure MDA-7/IL-24 protein will also be relevant in defining its anti-cancer activity. For studies using pure MDA-7/IL-24 protein, employing targeted delivery approaches, such as the use of polyfluorocarbonate microbubbles and ultrasound, as part of the ultrasound targeted microbubble destruction (UTMD) approach [28-30, 68-69] are also worth exploring. Based on MDA-7/IL-24's broad spectrum selective activity toward cancer cells and its multiplicity of anti-cancer effects, including “bystander” antitumor activity, anti-angiogenesis activity, direct cancer-selective apoptosis- and toxic autophagy-induction, immune modulatory activity and synergy with other therapeutic modalities, MDA-7/IL-24 holds significant promise for developing efficacious approaches for the therapy of both primary and metastatic breast cancers, as well as other types of cancer [10, 59].

## MATERIALS AND METHODS

### Adenoviruses

The tumor-specific conditionally replicating type 5 adenovirus expressing MDA-7/IL-24 (designated cancer terminator virus - *CTV*) was generated as described previously [20, 23, 70]. Briefly, the minimally active region of the tumor-specific PEG (progression elevated gene-3) promoter drives expression of E1A and the CMV promoter drives expression of MDA-7/IL-24 to generate a tumor-specific conditionally replicating adenovirus.

### Generation of transgenic mice

The Institutional Animal Care and Use Committee (IACUC) at Virginia Commonwealth University approved all animal studies. Female mice were used for all experiments. Food and water was provided *ad libitum*.

#### MMTV-PyMT transgenic mice

MMTV-PyMT transgenic mice expressing the potent transforming protein Polyoma virus middle tumor antigen (PyMT) under the transcriptional control of the MMTV LTR (mouse mammary tumor virus long terminal repeat) promoter were originally described by Guy et al. [4], and were purchased from The Jackson Laboratory (Stock #002374). A colony was established and maintained in our animal facility by breeding MMTV-PyMT hemizygous male mice to wild type FVB/N female mice. Mice were genotyped by PCR using sense primer (5′-TCCACTACACGATGACTACTGGTC-3′) and anti-sense primer (5′- ATGAGCTGGGGTACTTGTTCCTC-3′).

#### MMTV-*MDA-7* transgenic mice

To generate the MMTV-*MDA-7* transgene construct, a 2.3-kb BamHI fragment containing the MMTV LTR promoter from pRD812 [71] was inserted into the unique BamHI site of the pBSpKCR3 vector [72], which contains the rabbit β-globin intron 2 flanked by a portion of exon 2 and all of exon 3, including the polyadenylation site. A 0.6-kb MfeI fragment containing the human *mda-7/IL-24* cDNA was then inserted into the unique EcoRI site in β-globin exon 3. A 4.2-kb injection fragment was excised from the MMTV-*MDA-7* construct with XhoI, and transgenic mice were generated by standard methods in an FVB/N genetic background [73]. Potential founders were screened for the presence of the MMTV*-MDA-7* transgene by PCR analysis of genomic tail DNA using a rabbit β-globin intron 2 sense primer (5′-ACTACACCCTGGTCATCATCCTGC-3′) and a human *MDA-7/IL-24* cDNA anti-sense primer (5′-TGTGGACAAGGTAACAGCTCTCAG-3′). Amplification of DNA from mice carrying the transgene generated a 533-bp PCR product. Two independent founders were obtained, and line 2 was selected for use in the studies described here, based upon robust transgene expression in the mammary glands of pregnant and lactating female mice.

#### MMTV-*MDA-7*/MMTV-*Erbb2* compound transgenic mice

MMTV-*Erbb2* transgenic mice expressing the activated rat *c-neu* oncogene (*Erbb2*) under the transcriptional control of the MMTV LTR promoter were originally described by Muller et al. [3], and homozygous breeding pairs were obtained from The Jackson Laboratory (stock #005038) and maintained as homozygotes. To generate compound transgenic mice carrying both the MMTV*-MDA-7* and MMTV*-Erbb2* transgenes, hemizygous MMTV-*MDA-7* (line 2) mice (male or female) were mated to homozygous MMTV-*Erbb2* mice (male or female), so that all of the offspring were obligate hemizygotes for MMTV-*Erbb2*. Half of the offspring also carried the MMTV-*MDA-7* transgene, while the other half were MMTV-*MDA-7*-negative. Female offspring were therefore genotyped for the presence of the MMTV*-MDA-7* transgene.

### Treatment of MMTV-PyMT mice with an adenovirus expressing MDA-7/IL-24 (Ad5-*CTV*)

MMTV-PyMT mice develop mammary tumors spontaneously in all the mammary glands within a period from 2-3 months of age. A control group of 5 mice were left untreated and tumors were monitored in these mice. To determine the potential tumor suppressive effects of MDA-7/IL-24, Ad5-*CTV* or Ad5-E1A (1 × 10^8^ IU of the respective adenovirus in 50 μl) were injected intratumorally once a palpable tumor was observed in any mammary gland. As the mice developed tumors in the other mammary glands, at least 50% of the tumors that formed were injected with the respective adenovirus (e.g., 1 tumor was injected in mice with 1 or 2 tumors, 2 tumors were injected in mice with 3 or 4 tumors and so on). Care was taken to log the exact tumor that was injected to ensure repeated injection of the same tumors. Approximately 50% of the tumors within a particular mouse were left untreated to determine the bystander anti-tumor properties of MDA-7/IL-24. Each injected tumor received a maximum of 10 injections (depending on when palpable tumors were observed) over a 4-week period. Mice were sacrificed, tumors were harvested, formalin-fixed, paraffin-embedded and sectioned, and immunohistochemistry was performed following standard procedures.

### Assessing expression of *MDA-7/IL-24* in MMTV-MDA-7 transgenic mice

Mammary glands from pregnant and lactating female MMTV*-MDA-7* transgenic mice were harvested and flash frozen in liquid nitrogen. Protein and RNA were extracted using standard procedures and the expression of MDA-7/IL-24 was assessed at the transcript and protein level. Real-time quantitative PCR was performed according to standard procedures as described previously [73]. *MDA-7/IL-24* and *Gapdh* primer probes were obtained from Life Technologies. Western blotting was performed as described previously [74]. MDA-7/IL-24 antibody was obtained from GenHunter and EF1α antibody was obtained from EMD Millipore.

### Generating MMTV-PyMT and MMTV-PyMT luc cells

Mammary tumors were harvested from MMTV-PyMT mice to develop mouse “patient-derived xenograft” (murine PDX); mPDX tumors, similar to human PDX tumors. The MMTV-PyMT tumors were cut into small pieces and digested using trypsin-EDTA to obtain single cells. The cells were washed in sterile PBS and then plated in DMEM media supplemented with 5% Pencillin/Streptomycin and 5% fetal bovine serum (FBS). The cells were allowed to attach and media was replenished to remove the unattached cells. The cells were passaged to obtain MMTV-PyMT (mPDX) tumor cells. The cells were injected in FVB mice to ensure that the cells retained their tumor-forming abilities. To enable tumor growth monitoring using bioluminescent imaging, MMTV-PyMT cells were transfected with a luciferase-expressing construct (pGL4.50, Invitrogen). MMTV-PyMT cells stably expressing luciferase were selected using hygromycin and were designated MMTV-PyMT luc (mPDX luc) cells.

### Tumor growth assessment in MMTV-*MDA-7* transgenic mice

Each MMTV-*MDA-7* (positive or negative/control/non-transgenic littermate) female mouse was housed continuously with one male mouse. About 20 days after the birth of the first litter, MMTV-PyMT luc cells (1 × 10^6^ cells in 50 μl) were injected into the 4^th^ mammary fat pad of 10 MMTV-*MDA-7* transgenic mice and 10 control (non-transgenic littermate) mice. Tumor growth was monitored using bioluminescent imaging. Mice were sacrificed before tumors reached the maximum allowed limit. Tumors were harvested, formalin-fixed, paraffin-embedded and sectioned, and immunohistochemistry was performed following standard procedures.

### Tumor growth assessment in MMTV-*MDA-7*/MMTV-*Erbb2* compound transgenic mice

Each MMTV-*MDA-7*/MMTV-*Erbb2* female mouse was housed continuously with one male mouse. MMTV-*MDA-7* negative/MMTV-*Erbb2* positive female littermates were used as controls and were also housed continuously with a male mouse. Ten female mice were assessed per group. The female mice were monitored for tumor onset and then tumor growth twice weekly. Mice were sacrificed before tumors reached the maximum allowed limit. Tumors were harvested, formalin-fixed, paraffin-embedded and sectioned for immunohistochemistry.

### Immunohistochemistry

Immunohistochemistry was performed according to standard protocols. Briefly, tumor sections were deparaffinized at 60°C for 1 hour, followed by rehydration, and antigen retrieval using citrate buffer and heating. Avidin and biotin blocking kits and Vectastain ABC complex kits were obtained from Vector Laboratories. The MDA-7/IL-24 antibody utilized was from GenHunter and the E1A antibody was obtained from Thermo Scientific. Secondary antibodies were obtained from Jackson Immunoresearch. The slides were counter-stained using hematoxylin. Following staining, slides were dehydrated and mounted using Vectashield mounting media (Vector Laboratories).

### Analyses of tumor-infiltrating immune cells

For analysis of T cell infiltration and activation, MMTV-PyMT mammary tumor tissues were digested with collagenase D (1mg/mL) and DNase I (100μg/mL), and cell suspensions were filtered through a 70 μm cell strainer as previously described [75-76]. The viable mononuclear cells were isolated using the Histopaque (Sigma-Aldrich) gradient, and analyzed using a FACScaliber (BD Biosciences). To determinate the activation of CD8^+^ T cells, single cell suspension prepared from PyMT mammary tumors were stimulated with PMA plus ionomycin in the presence of Golgi-stop for 6 hours, followed by intracellular staining for IFN-γ or granzyme B-producing CD8^+^ cells. Fluorochrome-conjugated mouse mAbs, including FITC-CD8 (53-6.7), APC-CD4 (GK1.5) and PE-IFN-γ (XMG1.2), as well as CD16/CD32 (2.4G2), isotype control rat IgG2b (RTK4530), and IgG1 (RTK2071) were purchased from BioLegend (San Diego, CA).

### Statistical analysis

Statistical analyses were performed using GraphPad Prism 5. Data is presented as mean ± SEM. Student's t-test and Kaplan Meier analysis were applied based on the statistical mandates or suggestions of each analysis.

## SUPPLEMENTARY MATERIAL FIGURES


