# Supplementary material for: MDA-7/IL-24 functions as a tumor suppressor gene in vivo in transgenic mouse models of breast cancer
Source: Oncotarget. 2015 Oct 12;6(35):36928–42. doi: 10.18632/oncotarget.6047 (PMC4741906; doi:10.18632/oncotarget.6047)
Supplement: Supplementary file 1 [file oncotarget-06-36928-s001.pdf]

MDA-7/IL-24 functions as a tumor suppressor gene *in vivo* in transgenic mouse models of breast cancer

Supplementary Material

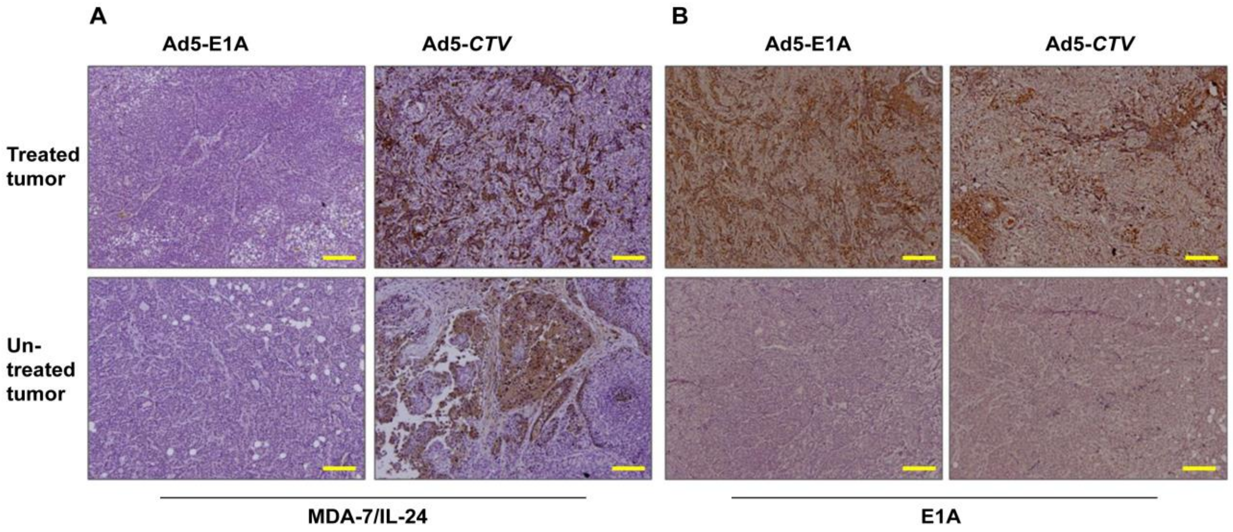

Supplemental Figure 1: MDA-7/IL-24 is expressed in both treated and untreated tumors within mice treated with Ad5-*CTV*. A. Immunohistochemistry showing expression of MDA-7/IL-24 in treated and untreated tumors of mice treated with Ad5-*CTV* but not in treated and untreated tumors of mice treated with Ad5-E1A. B. Immunohistochemistry showing expression of E1A in injected but not in uninjected tumors of mice treated with Ad5-E1A and Ad5-*CTV*. All images taken at 10X. Yellow bars = 100 μm.

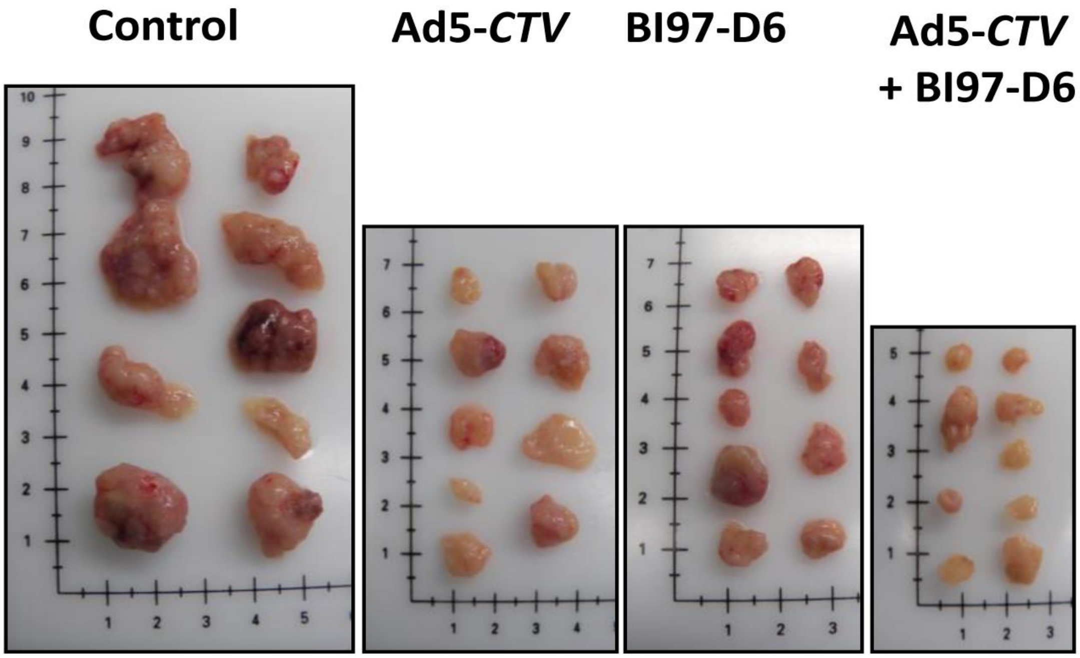

Supplemental Figure 2. MDA-7/IL-24 in combination with BI97-D6 causes further reduction in tumors in MMTV-PyMT mice. Representative images of total tumors present in MMTV-PyMT mice treated with Ad5-*CTV* and BI97-D6, singly and together, and untreated controls at the time of sacrifice. BI97-D6 (5 mg/kg) was administered intraperitoneally. Image dimensions are approximately adjusted to match the scale in each image.
